# Factors associated with poor outcomes in patients with severe acute respiratory infections in Bahrain

**DOI:** 10.1111/irv.13133

**Published:** 2023-04-26

**Authors:** Afaf Merza Mohamed, Adel Al Sayyad, Ebrahim Matar, Hasan M. Isa, Wafa Fawzi Hasan, Nawra Sayed Jalal Yusuf Hashim, Bayan Abduljalil Alajaimi, Qatrmeer Aldolabi

**Affiliations:** ^1^ Consultant Family Medicine & Public Health Ministry of Health Manama Bahrain; ^2^ Consultant Family Medicine, Epidemiology & Public Health, Chief of Disease Control Section, Ministry of Health. Associate Prof. of Family and Community Medicine CMMS, AGU Manama Bahrain; ^3^ Medical Intern, Eastern Health Cluster Dammam Saudi Arabia; ^4^ Consultant Pediatric Gastroenterologist, Pediatric Department Salmaniya Medical Complex, Arabian Gulf University Manama Bahrain; ^5^ Statistician, Salmaniya Medical Complex Manama Capital Bahrain; ^6^ Clinical attachment, Salmaniya Medical Complex Ministry of Health Manama Bahrain; ^7^ Public Health Specialist (PHS), Disease Control Section Ministry of Health Manama Bahrain

**Keywords:** Bahrain, influenza, outcome, respiratory infections, SARI

## Abstract

**Background:**

Severe acute respiratory tract infection (SARI) is a major global health threat. This study aimed to examine risk factors associated with poor outcomes in patients with SARI.

**Methods:**

All patients who met World Health Organization's (WHO) SARI case definition and were admitted to Salmaniya Medical Complex from January 2018 to December 2021 were included. Epidemiological and virological data were obtained and analyzed.

**Results:**

Of 1159 patients with SARI included, 731 (63.1%) patients were below 50 years, and 357 (30.8%) tested positive for viral pathogens. The most prevalent virus was Flu‐A (*n* = 134, 37.5%), SARS‐CoV2 (*n* = 118, 33%), RSV (*n* = 51, 14.3%), Flu B (*n* = 49,13.7%), other viruses (*n* = 3, 0.8%), and combined infection (*n* = 2, 0.6%). Six hundred fifty‐eight (56.8%) patients had comorbidities, mainly diabetes (*n* = 284, 43%) and heart disease ( *n* = 217, 33%). 183 (16%) patients were admitted to ICU, 110 (9%) needed mechanical ventilation, and 80 (7%) patients died.

The odds of ICU admission were higher for patients with hematological (OR 5.9, 95% CI 3.1–11.1) and lung diseases (OR 2.7, 95% CI 1.6–4.6). The odds of mechanical ventilation were higher among patients with lung disease (OR 3.1, 95% 1.7–5.5). The mortality odds were higher among patients above 50 (OR 2.4, 95% CI 1.4–4.1) and chronic kidney disease (OR 2.5, 95% CI 1.1–5.2).

**Conclusions:**

Being 50 years or above or having kidney, lung, or heart diseases was associated with worse SARI outcomes. Efforts and actions in developing better strategies to vaccinate individuals at high risk and early diagnosis and treatment should help in reducing the burden of SARI.

## 1 INTRODUCTION

Acute respiratory tract infections are one of the major global health threats. They have been the cause of more than one pandemic in the last century leading to the death of a large number of people. Back in 1918, the Spanish flu resulted in the death of around 50 million people, and in the 1950s and 60s, H2N2 (Asian flu) and H3N2 (Hong Kong flu) were estimated to cause the death of around 2 million people.^1^, ^2^, ^3^

Globally, severe acute respiratory infections (SARI) are estimated to be the cause of death in about 19% of all children younger than 5 years.^4^ In the United States alone, influenza viruses are predicted to be causing 12,000 to 61,000 deaths annually.^5^ Globally, it was also estimated that 291,000 to 645,000 deaths were caused by influenza viruses each year.^6^

In 1952, the influenza surveillance program was started by the World Health Organization (WHO). The aim of the influenza surveillance program is to reduce mortality and morbidity of the disease in the community by providing valuable information to the authorities for better interventions and control plans. In addition, it could be used to guide influenza vaccine production and detect emerging variants. Such programs are also important to prioritize intervention and give attention to high‐risk patients, where such infections could lead to higher rates of mortality.^7^

Many studies found that mortality was high among patients with SARI who were elderly or had associated comorbidities.^8^, ^9^, ^10^ However, risk factors associated with poor outcomes in SARI patients are not well established. During the H1N1 pandemic, mortality was high even among younger age groups.^11^ Accordingly, monitoring SARI in different age groups is essential to understand the spread of the disease in the community.^12^ Moreover, there were no published studies that addressed SARI in the Kingdom of Bahrain. Therefore, this study was conducted with the main objective of identifying the risk factors associated with poor outcomes, including mortality, ICU admission, and mechanical ventilation among patients admitted with SARI in Bahrain.

## 2 METHODS

### 2.1 Study design and study setting

This is a retrospective analysis of the SARI surveillance data in Bahrain, which was collected from January 2018 through December 2021. Data were gathered from all cases admitted with SARI in Salmaniya Medical Complex (SMC) during this period. In 2018, the SARI surveillance program was updated in the Kingdom of Bahrain. The main hospital in the country, SMC, was selected to be the sentinel site for SARI surveillance in Bahrain. Collected epidemiological and virological data were uploaded to the Eastern Mediterranean Flu Network (EMFLU), established by the WHO on a weekly basis.

### 2.2 Study population

All patients that met the definition of SARI by the WHO and as adopted by SARI surveillance in Bahrain were included in the study. Thus, patients admitted to the hospital due to acute respiratory tract infection, complaining of cough, and having a temperature of ≥38°C or a history of fever in the last 10 days were enrolled.^13^

### 2.3 Data collection

Data were collected and stored electronically in the WHO EMFLU database by an experienced public health specialist. Following data variables were collected as part of the surveillance: age, sex, area of residence, pre‐existing medical conditions, antiviral medications prescribed, influenza vaccination status, history of travel, history of contact with a sick patient, place of admission, admission to ICU, mechanical ventilation requirement, and status upon discharge (death or alive).

### 2.4 Specimen collection and testing

Influenza reverse transcription‐polymerase chain reaction (RT‐PCR) tests were performed for all the patients in the national influenza center in the public health laboratory to identify the causative agents. COVID‐19 PCR was also done during the coronavirus pandemic years 2020–2021. Integrated surveillance (SARS‐CoV2, influenza and RSV) was conducted using nasopharyngeal swabs. Specimens were stored in a viral transport media and refrigerated until processing within a period of 24 h from the time of sampling. Laboratory processing and diagnosis of specimens were performed according to the WHO standards.^14^

### 2.5 Statistical analysis

Patients' data were first exported from the EMFLU database into Microsoft Excel and transferred to IBM Statistical Package for the Social Sciences (SPSS) software version 21 for statistical analysis. Quantitative variables were reported as mean and standard deviations, whereas qualitative variables were described using counts and percentages to obtain the descriptive analysis. Categorical groups of variables were compared using the Chi‐square test. Univariate analysis and odds ratios were also calculated. Factors associated with an increased risk of mechanical ventilation, ICU admission, or death were assessed using logistic regression. Significant factors found through univariate logistic regression and relevant factors from the literature were analyzed using a multivariate logistic regression model to adjust for confounders. To examine the individual impact of each comorbidity on the risk of mortality, we analyzed the data using the Chi‐Square test, comparing patients with specific comorbidity with patients with no comorbidities (e.g., patients having diabetes compared with those without diabetes).

### 2.6 Ethical approval

This study was conducted in accordance with the principles of the Helsinki Declaration, and it was ethically approved by the Health Research Committee in the Ministry of Health, Kingdom of Bahrain. Each patient signed a written informed consent upon hospital admission, and the research team received the data anonymized from the influenza surveillance team.

## 3 RESULTS

### 3.1 Patients' characteristics

During the study period, a total of 1159 patients were found to be enrolled in the SARI surveillance program. Most of the patients were males (*n* = 674, 58.2%) and below the age of 50 years (*n* = 731, 63.1%). The mean age was 40 ± 25 years. The rate of influenza vaccination among the patients was low (*n* = 25, 2.2%). None of the vaccinated patients tested positive for influenza. Only 130 (11.2%) patients received antiviral medications.

Most of the admitted patients had underlying comorbidities (*n* = 658, 56.8%), with 320 (27.6%) having more than one comorbidity. The most common was diabetes mellitus (*n* = 284, 24.5%), followed by heart disease (*n* = 217, 18.7%) and asthma (*n* = 161, 13.9%) (Table 1).

**TABLE 1:** Epidemiological and virological characteristics, and outcome of 1159 enrolled severe acute respiratory infection patients, Bahrain, 2018–2021.

Patient characteristics			Total patients (*n* = 1159)		Ventilation		ICU admission		Death	
			Count	Column N %	OR (CI)	P value	OR (CI)	P value	OR (CI)	P value
Demographics	Sex	Male	674	58.2%	1.2 (0.8–1.8)	0.313	0.8 (0.6–1.1)	0.216	1.1 (0.7–1.8)	0.554
		Female	485	41.8%						
	Age	<50	731	63.1%	1.6 (1.1–2.4)	0.019	0.9 (0.7–1.3)	0.667	3.1 (1.9–4.9)	<0.001*
		≥50	428	36.9%						
Vaccination	Seasonal influenza vaccine	No	1134	97.8%	0.4 (0.1–2.9)	0.361	0.7 (0.2–2.4)	0.601	‐	‐
		Yes	25	2.2%						
Antiviral use	Antiviral use	No	1029	88.8%	2.2 (1.3–3.6)	0.003	2.3 (1.5–3.5)	<0.001*	1.3 (0.7–2.5)	0.458
		Yes	130	11.2%						
PCR results	RT‐PCR	Negative	802	69.2%	1.1 (0.7–1.7)	0.646	1.6 (1.1–2.2)	0.007	1.1 (0.7–1.8)	0.733
		Positive	357	30.8%						
	Total influenza viruses (A&B)^b^	No	976	84.2%	‐	‐	‐	‐	‐	‐
		Yes	183	15.8%						
	Influenza B	No	1110	95.8%	0.4 (0.1–1.6)	0.202	0.9 (0.4–2)	0.768	0.9 (0.3–2.9)	0.826
		Yes	49	4.2%						
	Total influenza A	No	1025	88.4%	1.7 (0.9–3.5)	0.122	1.3 (0.7–2.4)	0.405	1.6 (0.7–3.7)	0.243
		Yes	134	11.6%						
	Influenza A H1N1	No	1092	94.2%	1.1 (0.5–2.5)	0.783	2.1 (1.2–3.6)	0.012	0.6 (0.2–2)	0.424
		Yes	67	5.8%						
	Influenza A H3N2	No	1151	99.3%	1.5 (0.8–2.5)	0.182	1.7 (1.1–2.7)	0.014	1.1 (0.6–2.2)	0.786
		Yes	8	0.7%						
	Influenza A unsub typed	No	1100	94.9%	1.1 (0.7–1.9)	0.654	1.5 (1–2.2)	0.045	1 (0.6–1.9)	0.907
		Yes	59	5.1%						
	Respiratory syncytial virus	No	1108	95.6%	2.1 (1–4.5)	0.047	1.3 (0.7–2.7)	0.446	1.2 (0.4–3.3)	0.787
		Yes	51	4.4%						
	SARS‐CoV2	No	1041	89.8%	0.7 (0.3–1.4)	0.292	1.4 (0.9–2.3)	0.154	1.1 (0.5–2.3)	0.743
		Yes	118	10.2%						
	Other viruses^a^	No	1156	99.7%	‐	‐	‐	‐	‐	‐
		Yes	3	0.3%						
	Mixed infections (Influenza B + Covid19)	No	1157	99.8%	‐	‐	‐	‐	‐	‐
		Yes	2	0.2%						
Underlying conditions	Comorbidities	No	501	43.2%	1.8 (1.2–2.7)	0.007	1.9 (1.4–2.7)	<0.001*	3 (1.7–5.2)	<0.001*
		Yes	658	56.8%						
	Diabetes	No	875	75.5%	1.3 (0.8–2)	0.241	0.8 (0.5–1.1)	0.201	1.5 (0.9–2.5)	0.087
		Yes	284	24.5%						
	Asthma	No	998	86.1%	0.7 (0.3–1.3)	0.218	0.6 (0.4–1.1)	0.086	0.7 (0.3–1.4)	0.300
		Yes	161	13.9%						
	Heart disease	No	942	81.3%	1.8 (1.2–2.8)	0.008	1.6 (1.1–2.4)	0.009	2.4 (1.5–3.9)	<0.001*
		Yes	217	18.7%						
	Other comorbidities^a^	No	978	84.4%	2.4 (1.5–3.7)	<0.001*	1.6 (1.1–2.4)	0.022	‐	‐
		Yes	181	15.6%						
	Chronic liver disease	No	1149	99.1%	‐	‐	‐	‐	3.4 (0.7–16.4)	0.123
		Yes	10	0.9%						
	Chronic hematological disorder	No	1115	96.2%	1 (0.3–2.7)	0.926	4.4 (2.4–8.2)	<0.001*	0.6 (0.2–2.7)	0.533
		Yes	44	3.8%						
	Immune compromised	No	1094	94.4%	1 (0.4–2.3)	0.941	0.7 (0.4–1.6)	0.430	2.3 (1.1–4.9)	0.027
		Yes	65	5.6%						
	Chronic lung disease	No	1076	92.8%	3.2 (1.8–5.6)	<.001*	2.5 (1.5–4.1)	<0.001*	2.2 (1.1–4.4)	0.021
		Yes	83	7.2%						
	Neuromuscular dysfunction	No	1124	97.0%	0.9 (0.3–3)	0.851	1.1 (0.5–2.7)	0.824	0.8 (0.2–3.4)	0.779
		Yes	35	3.0%						
	Chronic kidney disease	No	1103	95.2%	2.5 (1.2–4.9)	0.010	2.2 (1.2–4.1)	0.009	4.2 (2.1–8.2)	<0.001*
		Yes	56	4.8%						
	>1 Comorbidity	No	839	72.4%	1.8 (1.2–2.7)	0.005	1.3 (0.9–1.8)	0.179	2.7 (1.7–4.3)	<0.001*
		Yes	320	27.6%						

Odds ratios were not calculated for some variables due to low numbers.

^a^ Other viruses and comorbidities were not specified or recorded other than the ones listed above, thus not included in the analysis.

^b^ Analysis was not conducted for total influenza viruses' results, each subtype was analyzed separately.

* Significant association at the 0.05 level (2‐tailed).

### 3.2 Laboratory results

The RT‐PCR test showed that 357 (30.8%) patients had positive results. The detected viruses were influenza in 183 (15.8%), SARS‐CoV2 in 118 (10.2%), and respiratory syncytial viruses (RSV) in 51 (4.4%) patients. Influenza A virus was identified in 134 (11.6%) and influenza B in 49 (4.2%) patients. Two patients (0.2%) had combined influenza and SARS‐CoV2 infection, and 3 (0.2%) had other viruses.

### 3.3 Admission to ICU

ICU admission was noted in 183 (15.8%) patients; 114 (62.3%) of them were males, and 118 (64.5%) were below 50 years. The majority of ICU‐admitted patients did not receive the seasonal influenza vaccine (*n* = 180, 98.4%) nor an antiviral medication (*n* = 147, 80.3%). Seventy‐two (39.3%) patients tested positive for a viral pathogen, 31 (16.9%) of them for influenza A viruses and 24 (13.1%) for SARS‐CoV2 (Table 1). One hundred twenty‐seven (69.4%) patients had comorbidities, 47 (25.7%) had heart diseases, 25 (13.7%) had chronic lung diseases, 19 (10.4%) had chronic hematological disorders, and 16 (8.7%) patients had chronic kidney diseases.

Further analysis using univariate logistic regression revealed that SARI patients who had comorbidities were more likely to be admitted to ICU (OR 1.9 [95% CI 1.4–2.7], *P* < 0.001). Patients with chronic hematological diseases (OR 4.4 [95% CI 2.4–8.2], *P* < 0.001), chronic lung diseases (OR 2.5 [95% CI 1.5–4.1], *P* < 0.001), chronic kidney diseases (OR 2.2 [95% CI 1.2–4.1], *P* = 0.009), and chronic heart diseases (OR 1.6 [95% CI 1.1–2.4], *P* = 0.009) were at higher risk for ICU admission. Patients who tested positive for influenza A (OR 1.7 [95% CI 1.1–2.7], *P* = 0.014) and those given antiviral medication were more likely to be admitted to the ICU (OR 2.3 [95% CI 1.5–3.5], *P* < 0.001) (Table 1).

### 3.4 Mechanical ventilation

Mechanical ventilation was required in 110 (9.5%) patients, 59 (53.6%) of them were males, 52 (47.2%) were above 50 years, 22 (20%) had received antiviral medications during their hospital stay, and only one (0.9%) patient reported to be vaccinated. Of the ventilated patients, 36 (32.7%) tested positive for a viral pathogen; the commonest was influenza A (*n* = 17, 15.4%), followed by RSV (*n* = 9, 8%). The majority had at least one type of comorbidities (*n* = 76, 69%), and 43 (39%) had multiple morbidities. Diabetes mellitus was the most frequent comorbidity (*n* = 32, 29.1%), followed by chronic heart disease (*n* = 31, 28.2%).

The results of univariate logistic regression for factors associated with mechanical ventilation are shown in Table 1. Patients older than 50 years (OR 1.6 [95% CI 1.1–2.4], *P* = 0.019), and those with comorbidities (OR 1.8 [95% CI 1.2–2.7], *P* = 0.007) were at a higher risk. Risk of mechanical ventilation was specifically higher among patients with chronic lung disease (OR 3.2 [95% CI 1.8–5.56], *P* < 0.001), chronic kidney disease (OR 2.5 [95% CI 1.2–4.9], *P* = 0.01), and those with chronic heart diseases (OR 1.8 [95% CI 1.2–2.8], *P* = 0.008). Patients with RSV were at a higher risk of being ventilated (OR 2.1 [95% CI 1–4.5], *P* = 0.047). Administration of an antiviral medication was also linked to higher risk (OR 2.2 [95% CI 1.3–3.6], *P* = 0.003).

### 3.5 Case fatality

The case fatality of SARI was 6.2% (80/1159). The mean age at death was 52 ± 26 years, and most were males (*n* = 44, 55%). Twenty‐six (32.5%) deceased patients had a positive virological test. The most common pathogen identified among them was influenza A in 10 (12.5%) patients followed by COVID‐19 in nine (11.3%) patients. Antiviral medications were given to 11 (13.8%) patients, and none of them had received the influenza vaccine in the season. Sixty‐three (78.8%) patients had comorbidities, and 39 (48.8%) of them had more than one comorbidity. The most common was heart disease, 27 (33.8%), followed by diabetes in 26 (32.5%) patients (Table 1).

On logistic regression analysis, higher mortality was noted in patients older than 50 years (OR 3.1 [95% CI 1.9–4.9], *P* < 0.001), especially those with underlying chronic disease (OR 3 [95% CI 1.7–5.2], *P* < 0.001). Specifically, having chronic kidney disease (OR 4.2 [95% CI 2.1–8.2], *P* < 0.001), chronic heart diseases (OR 2.4 [95% CI 1.5–3.9], *P* < 0.001), chronic lung diseases (OR 2.2 [95% CI 1.1–4.4], *P* = 0.021), or being immunocompromised (OR 2.3 [95% CI 1.1–4.9], *P* = 0.027) were linked to higher mortality (Table 1).

Case fatality was significantly associated with diabetes (*P* < 0.001), chronic heart (*P* < 0.001), kidney (*P* < 0.001), neuromuscular diseases (*P* < 0.001), immunocompromised patients (*P* < 0.001), and those with liver disease (*P* = 0.005) when compared with patients with no comorbidities.

### 3.6 Logistic regression/multivariate analysis

After adjusting for confounders using multivariate logistic regression models, ICU admission was linked to having hematological diseases (aOR 5.9 [95% CI 3.1–11.1], *P* < 0.001), lung diseases (aOR 2.7 [95% CI 1.6–4.6], *P* < 0.001), or kidney diseases (aOR 2.2 [95% CI 1.2–4.2], *P* = 0.016), receiving antiviral medication (aOR 2.5 [95% CI 1.56–3.9], *P* < 0.001) or having chronic heart disease (aOR 1.5 [95% CI 1.0–2.3], *P* = 0.035).

A higher risk of mechanical ventilation was associated with having chronic lung diseases (aOR 3.1 [95% CI 1.7–5.5], *P* < 0.001), infection with RSV (aOR 2.5 [95% CI 1.1–5.5], *P* = 0.02), antiviral medication use (aOR 2.3 [95% CI 1.3–3.9], *P* < 0.001), or kidney disease (aOR 2.1 [95% CI 1.0–4.4], *P* = 0.05). A higher risk of mortality was associated with patients aged above 50 years (aOR 2.4 [95% CI 1.4–4.1], *P* < 0.001), and with those having chronic kidney disease (aOR 2.4 [95% CI 1.1–5.2], *P* = 0.02) (Table 2).

**TABLE 2:** Characteristics and level of association with mortality, ICU admission, and mechanical ventilation in patients with a severe acute respiratory infection.

Outcome	Variable	Multivariate analysis	
		OR (CI)	P value
Mortality	Age	2.4 (1.4–4.1)	<0.001*
	Heart disease	1.5 (0.9–2.6)	0.124
	Immune compromised	2 (0.9–4.3)	0.100
	Chronic lung disease	1.8 (0.9–3.7)	0.098
	Chronic kidney disease	2.4 (1.1–5.2)	0.020*
	Influenza B	1.1 (0.3–3.7)	0.908
	H1N1	1.9 (0.8–4.5)	0.136
	Influenza A	0.5 (0.1–1.6)	0.237
	Respiratory syncytial virus	1.5 (0.5–4.5)	0.492
ICU admission	Antiviral medication	2.5 (1.6–3.9)	<0.001*
	Influenza A	1.7 (0.9–3.2)	0.074
	Heart disease	1.5 (1–2.3)	0.035*
	Chronic hematological disorder	5.9 (3.1–11.1)	<0.001*
	Chronic lung disease	2.7 (1.6–4.6)	<0.001*
	Chronic kidney disease	2.2 (1.2–4.2)	0.016*
	Influenza B	1.3 (0.6–3.1)	0.483
	H1N1	1.3 (0.7–2.4)	0.492
	Respiratory syncytial virus	1.6 (0.8–3.4)	0.194
Mechanical ventilation	Age	1.2 (0.7–1.8)	0.510
	Antiviral medication	2.3 (1.3–3.9)	<0.001*
	Influenza A	1.2 (0.7–2.2)	0.520
	Respiratory syncytial virus	2.5 (1.1–5.5)	0.02*
	Chronic lung disease	3.1 (1.7–5.5)	<0.001*
	Chronic kidney disease	2.1 (1–4.4)	0.05*
	Heart disease	1.5 (0.9–2.5)	0.120

* Significant association at the 0.05 level (2‐tailed).

## 4 DISCUSSION

Whereas most of our enrolled SARI patients were adults, a similar study that was done in Egypt had mostly children. The mean age of our patients was 40, whereas, in Egypt, the mean age was 6 years.^8^ However, a study conducted in Arizona had a higher mean age of 63 years.^12^ Most SARI cases had comorbidities. This finding was in line with findings from other SARI surveillance studies conducted in Egypt, Chile, and Arizona.^8^, ^12^, ^15^ The most prevalent diseases were diabetes and chronic heart disease among adults in SARI surveillance. This could be explained by the high prevalence of both diseases in the general population.^16^, ^17^ In Bahrain, the prevalence of diabetes and hypertension is high, at 15% and 33%, respectively.^18^

### 4.1 Laboratory results

All of the enrolled patients were tested for respiratory pathogens via RT‐PCR of samples collected by nasopharyngeal swabs. However, only 30% had a causative agent detected. This was similar to findings in five Eastern Mediterranean countries, Vietnam, and Arizona.^8^, ^9^, ^12^, ^19^ In a multicentric European study, the causative agent's detection rate in SARI surveillance ranged from 2.1% to 100%.^10^ The low yield of positive results could be due to the delay of collection of specimens, as it is recommended to be within 4 days from the onset of illnesses, or due to other respiratory pathogens not being tested for.^20^ A study in India on SARI cases among children found that most positive cases were related to human metapneumovirus followed by influenza viruses.^21^ SARI cases in Bahrain are not routinely tested for all respiratory viruses.

### 4.2 ICU admission

The ICU admission rate in this study was lower (15.8%) than those reported by other studies, which ranged from 25% in Egypt to 40% in Arizona.^8^, ^12^, ^15^, ^22^ The lower rate of admission in Bahrain might be related to the lower age of patients with SARI compared with the study from Arizona.

Our study found that comorbidities increased the risk of ICU admission. Similar results were found in other studies in the Eastern Mediterranean region, the European region, and Chile.^9^, ^10^, ^15^, ^22^ In the European region study, further analysis found that chronic heart and lung diseases were linked to a higher risk of admission to the ICU.^10^

Furthermore, patients with SARI were at higher risk of being admitted to ICU if they tested positive for influenza A. In this study, 31 (16.9%) of the ICU‐admitted patients tested positive for influenza A. Yet, findings in the literature were inconsistent; whereas some linked the detection of a causative agent with lower rates of admission,^8^, ^12^ others found no association between testing positive and ICU admissions.^9^ However, one study found a higher risk of admission in influenza‐positive patients, similar to our findings.^10^

### 4.3 Mechanical ventilation

Pneumonia is the commonest complication of influenza infections.^23^ Susceptible patients may deteriorate to reach respiratory failure.^5^ One hundred ten (9.5%) patients developed respiratory failure during their SARI episode, which required mechanical ventilation. This rate was lower than those in other surveillance programs, which ranged from 19% to 22%.^8^, ^12^, ^15^ The majority of ventilated patients had chronic diseases (*n* = 76, 69%), and no viral agent was detected in 74 (67.3%) patients.

Although data on the risk factors resulting in a SARI patient requiring mechanical ventilation is scarce, our results were comparable to findings from a study conducted in our (EMR) region.^9^

RSV infections were linked with a milder form of the disease in children with SARI^8^; despite that, we found that it was associated with higher rates of ventilation when adults were infected.

### 4.4 Case fatality

Eighty (6.2%) of the 1159 enrolled patients have died. Most of them were above 50 years (62.5%), similar to the results of a study from Arizona.^12^ Moreover, the majority had comorbidities (78.8%), comparable to findings from Europe, Arizona, and Egypt.^8^, ^12^, ^22^ The mortality rate was found to be on the lower side compared with other studies, which ranged from 2.2% in Egypt to 15% in Europe.^8^, ^12^, ^15^, ^22^ A higher mortality rate compared with patients from Egypt could be explained by the younger ages included in the Egyptian surveillance study.

Higher rates of mortality were correlated with age above 50 years or having underlying diseases. Studies in the region found similar results; those aged above 50 years and having underlying diseases increased the odds of severe outcomes (indicated by death, ICU admission, or ventilation).^8^, ^9^ The study done in Europe, along with a globally conducted study, revealed similar results.^10^, ^24^ However, the study conducted in Chile found that age and comorbidities were not linked with higher mortality rates.^15^

We found that an immunocompromised state and chronic lung, kidney, or heart diseases were all associated with higher mortality. This is comparable to the European study findings.^10^

Detecting a viral pathogen in our admitted patients was not associated with increased mortality. Likewise, many studies had similar findings.^8^, ^9^, ^15^ However, a study conducted in Europe reported the influenza virus as a risk factor for mortality.^10^ This may suggest that factors other than the causative agents, such as patient characteristics, age, and comorbidities, played a more significant role in the outcome of patients with SARI.

### 4.5 Vaccination

The seasonal influenza vaccine remains the mainstay measure to decrease the burden of SARI by preventing influenza infections and lowering the need for ICU admission and mortality in infected patients.^25^, ^26^, ^27^ In Bahrain, the influenza vaccine is recommended for all citizens, below the age of 5, above the age of 50, or having chronic diseases.^28^ This meant that 871 (75%) of our patients should have been vaccinated, yet only 25 (2%) got the vaccine. None of the vaccinated patients tested positive for influenza viruses.

### 4.6 Antiviral treatment

Studies found that prompt treatment of influenza infections with antiviral drugs decreases the risk of mortality and reduces hospital length of stay.^29^, ^30^, ^31^, ^32^ Furthermore, it is associated with lower hospitalization rates in high‐risk patients.^33^ Timely initiation of antiviral treatment is key, especially in high‐risk groups.^29^, ^31^, ^34^ Despite that, only 130 (11.2%) of our admitted patients received antiviral medication. In our sample, patients taking the antiviral medication were more likely to be admitted to the ICU or ventilated. As data about the time of initiation of treatment from the onset of symptoms was not available, we suspect that these findings were due to the administration of antivirals in critically ill patients, after the progression of the disease and in non‐influenza patients.

### 4.7 Limitation

The study used the SARI surveillance data, which helps to identify the limitations of the surveillance system.^35^ Examples of these limitations are incomplete data collection, inaccuracies, and misdiagnosis. In 181 (15.6%) patients, comorbidities were recorded as “others” but were not specified in the original data set. Moreover, 44% of influenza A‐positive samples were not subtyped, which could improve the analysis. Additionally, this study did not investigate the relationship between patients' outcomes and presenting symptoms or clinical course. Whereas this study focused on risk factors associated with poor outcomes regardless of the test result, most of the previous studies have focused on patients with detected pathogens, mainly seasonal influenza and H1N1 pdm viruses. Thus, the results of each study should be interpreted within their own context. On the other hand, this study has its own promise; it is the first study to address risk factors of severe outcomes of SARI in patients from Bahrain and covers 4 years of surveillance data. In our study, all patients enrolled in the surveillance program were tested for viral infections. Furthermore, choosing SMC as the only sentinel site in Bahrain receiving patients from all areas and all ages in the kingdom ensured the homogeneity and representativeness of our sample size.

### 4.8 Recommendation

Surveillance programs are extremely crucial to help monitor and evaluate patterns of emerging diseases and develop strategies and interventions. Moreover, patients at risk should be identified in outpatient settings for prompt testing, and the use of antivirals may hinder disease progression.^34^ Improving vaccination rates using different approaches should be considered to increase coverage, especially in high‐risk individuals such as the elderly and patients with comorbidities. Vaccination strategies such as sending reminders to susceptible patients and their healthcare providers should be seen as an effective option and may be adopted.^36^, ^37^

## 5 CONCLUSIONS

Severe acute respiratory infections are a serious burden on any healthcare system and lead to high morbidities and mortalities. This study indicates that patients above 50 years and those with comorbidities were at higher risk for poor outcomes. One‐third of the patients had identifiable pathogens. Influenza A was linked with higher rates of ICU admission, and RSV was linked with a higher risk of mechanical ventilation. Further measures to prevent severe acute respiratory infections should be taken, and efforts to develop better strategies regarding the vaccination of high‐risk individuals should be considered. Further studies addressing the gaps identified in this study may provide additional evidence on this important global health issue.

## AUTHOR CONTRIBUTIONS

**Afaf Merza Mohamed:** Conceptualization; data curation; formal analysis; investigation; methodology; project administration; resources; software; supervision; validation; visualization; writing–original draft; writing–review and editing. **Adel Al Sayyad:** Formal analysis; methodology; supervision; writing–review and editing. **Ebrahim Matar:** Formal analysis; investigation; project administration; resources; software; supervision; writing–original draft. **Hasan M. Isa:** Conceptualization; methodology; writing–original draft; writing–review and editing. **Wafa Fawzi Hasan:** Formal analysis; investigation; software. **Nawra Sayed Jalal Yusuf Hashim:** Formal analysis; methodology; writing–original draft. **Bayan Abduljalil Alajaimi:** Formal analysis; methodology; writing–original draft. **Qatrmeer Aldolabi:** Conceptualization; data curation; resources.

## CONFLICT OF INTEREST STATEMENT

No financial or non‐financial benefits have been received or will be received from any party directly or indirectly related to this article's subject.

### PEER REVIEW

The peer review history for this article is available at https://www.webofscience.com/api/gateway/wos/peer-review/10.1111/irv.13133.

## ETHICS STATEMENT

This study was conducted in accordance with the principles of the Helsinki Declaration, and it was ethically approved by the Health Research Committee in the Ministry of Health, Kingdom of Bahrain.

## Data Availability

Data will be made available on reasonable request.
